# Study on Regional Differences and Convergence of Green Development Efficiency of the Chemical Industry in the Yangtze River Economic Belt Based on Grey Water Footprint

**DOI:** 10.3390/ijerph19031703

**Published:** 2022-02-02

**Authors:** Yunbo Xiang, Wen Shao, Shengyun Wang, Yong Zhang, Yaxin Zhang

**Affiliations:** 1 School of Architecture and Art Design, Hunan University of Science and Technology, Xiangtan 411201, China; yunb.xiang@hnust.edu.cn (Y.X.); shaowen0921@163.com (W.S.); hnkdzhyong@sina.com (Y.Z.); 2 Research Center for Economic and Social Development in Central China of Nanchang University, Nanchang University, Nanchang 330031, China; zhangyaxin0926@163.com

**Keywords:** chemical industry, green development efficiency, grey water footprint, regional differences, convergence, Yangtze River Economic Belt

## Abstract

Grey water footprint is included in the green development efficiency evaluation index system of the chemical industry. From 2002 to 2016, the super efficiency Slack Based Measure (SBM) model was used to measure the green development efficiency of the chemical industry in the Yangtze River Economic Belt. Dagum Gini coefficient and its decomposition method were used to decompose the regional differences of green development efficiency of the chemical industry in the Economic Belt, and the coefficient of variation method and panel data regression model were used to test the convergence characteristics. The following results were obtained. (1) The total grey water footprint of the chemical industry in the Yangtze River Economic Belt showed a fluctuating downward trend from 2002 to 2016. (2) The green development efficiency of the chemical industry in the Yangtze River Economic Belt was significantly improved, and the spatial differentiation law of gradient decline in the upper, middle, and lower reaches of the Economic Belt was shown. (3) The regional difference of green development efficiency of the chemical industry in the Yangtze River Economic Belt initially showed an expanding trend and then a narrowing trend. Regional differences in the upper reaches of the Yangtze River increased while those in the middle reaches first increased and then decreased, whereas those in the lower reaches decreased significantly. The variance in green development efficiency of the chemical industry is the main cause of regional differences. (4) From 2012 to 2016, the Yangtze River Economic Belt had obvious convergence in its whole region, middle reaches, and lower reaches and an inconspicuous convergence in the upstream area. Regional difference of green development efficiency of the chemical industry in the Economic Belt was the combined effect of the results of environmental regulation, industrial structure, foreign investment intensity, and scientific and technological advancements. Our results have high theoretical reference values and practical guiding significance for implementing the green efficiency promotion strategy of the chemical industry in Yangtze River Economic Belt by region and classification.

## 1. Introduction

The Yangtze River Economic Belt is one of the most critical contradiction areas between economic development and environmental protection in China [1]. A total 40% of available freshwater resources and more than 20% of its wetland resources in China are concentrated in the Yangtze River Basin, which covers 204 national aquatic germplasm resources protection zones. The River Basin is one of the important ecological security barriers and economic centers in China [2]. The chemical industry is a basic and pillar industry of the national economy, with high dependence on water and energy as well as high safety and environmental risks [3]. China is the largest chemical producer in the world. In 2018, its chemical turnover was 119.8 million EUR, accounting for 35.8% of the global chemical sales for the year [4]. The production value of chemical products in the Yangtze River Economic Belt accounts for more than 40% of the country’s total. At present, “chemical industry encircling the river” poses challenges to the Yangtze River Economic Belt. There are more than 400,000 chemical enterprises, 5 steel bases, 7 oil refineries, and many large petrochemical bases along the Yangtze River, leading to a greater risk of environmental pollution [5]. In the “joint efforts to protect” and under the general requirements of “no large-scale development”, the green transformation and development of the chemical industry in the Yangtze River Economic Belt is particularly urgent.

The Chinese economy has entered a high-quality development stage from the high-speed growth stage now. The traditional industrial development mode of high energy consumption, high pollution, and high emission has gradually changed to the intensive, efficient, and sustainable green development mode. The Chinese government has attached great importance to the green development of the chemical industry in the Yangtze River Economic Belt. On 14 November 2020, General Secretary Xi Jinping presided over a forum that aimed to promote the comprehensive development of the Yangtze River Economic Belt, stressing the need to make it the main battlefield of Chinese ecological priority and green development. Relevant departments of the state have also issued a series of policies and regulations, such as guiding opinions on strengthening the green development of industries in the Economic Belt, the ecological environment protection plan for the area, the Law of the People’s Republic of China on the Protection of the Yangtze River, and so forth. All of these endeavors actively promote the green transformation and upgrading of the chemical industry in the Yangtze River Economic Belt. In the past five years, more than 8000 chemical enterprises along the Economic Belt have been reformed, relocated, transformed, or closed. Remarkable achievements have been made in the green transformation and development of the chemical industry. The ecological environment has been significantly improved. The proportion of excellent water quality sections in the Yangtze River basin increased from 82.3% in 2016 to 91.7% in 2019 and further increased to 96.3% from January to November 2020. The elimination of poor V water bodies achieved for the first time in 2020. It can be seen that comprehensively promoting the green development of the chemical industry and improving its green development efficiency [6] are key to solving the dilemma of the “chemical industry surrounding the river”, which ensures environmental and industrial development safety and realizes the sustainable development of the chemical industry. The Yangtze River Economic Belt includes 9 provinces, 2 cities, and 11 provincial administrative units and covers an area of approximately 2.05 million km^2^ [7]. Due to the differences in resource conditions, economic development levels, innovation abilities, and chemical industry development histories, the spatial distribution and green development level of the chemical industry show spatial heterogeneity, which increases the challenge for the Economic Belt to promote the industrial green development. In order to measure the gray water footprint and green development efficiency of the chemical industry in the Yangtze River Economic Belt, to reveal the spatial differences and their convergence in the green development efficiency of the chemical industry in 11 provinces and cities, and to provide policy support for the green development of the chemical industry in the Yangtze River Economic Belt, this research systematically studied the regional differences and convergence of green development efficiency of the chemical industry in the Yangtze River Economic Belt. The results are expected to be valuable in theoretical reference and practical significance for implementing green development promotion strategy of the chemical industry in different regions and categories.

## 2. Literature Review

The concept of “green development” was first proposed by the United Nations Development Programme in 2002. The essence of green development is to regard resources and the environment as endogenous factors of growth and provide a balance between economic growth and ecological environment protection by changing the dynamic mechanism of economic development to form a new sustainable development model [8,9]. From the perspective of input and output, green development efficiency refers to the proportional relationship between green development output and input. Green development efficiency is an important indicator to analyze the degree of green development of industries and is often used to reflect the completion degree, achievements, and effectiveness of green development. Since data envelopment analysis (DEA) can consider a variety of input and output and does not need to set specific function forms, it has become the mainstream method to measure green development efficiency [10]. Pittman (1983) first included “undesirable” output into the productivity analysis process [11]. Chung et al. (1997) proposed directional distance function and the Malmquist–Luenberger Index (MLI), which carries out productivity evaluation after the “undesirable” output is considered reasonable [12]. Tone (2001, 2002) proposed a SBM model that considers relaxation measures to effectively overcome radial and angular defects [13,14]. A DEA analysis method based on the measurement of slack variables, which puts the input and output slack directly into the objective function so that it can directly measure the inefficiency caused by slack compared to the optimal production frontier, thus solving the problem of input and output slack in the traditional DEA model, removing the inefficiency caused by slack, and also solving the problem of productivity evaluation in the presence of non-expected outputs. Many scholars used the DEA model to discuss the green development efficiency of the chemical industry. Tanzil and Beloff (2006) summarized the sustainability indicators and indicators of the chemical industry, focusing on ecological efficiency and company-specific indicators [15]. Alessandro et al. (2017) measured the environmental economic efficiency of Italian and German chemical enterprises [16]. Yeh Jiahuey et al. (2019) calculated the total factor green energy efficiency of China’s chemical industry [17]. Yijun Zhang et al. (2020) used the three-stage SBM–DEA model and MLI to measure the green total factor productivity (GTFP) of China’s chemical industry [6]. Sun Honghai (2017) used super-efficiency DEA to calculate the ecological efficiency of 25 petrochemical enterprises in China [18]. Yuan Yaqiong (2018) used DEA and value-driven analysis to evaluate the ecological efficiency of heavy chemical enterprises in Beijing, Tianjin, and Hebei region from 2012 to 2016 [19]. Lu Qiuqin et al. (2020) used the improved three-stage DEA model to evaluate the transformation and upgrading efficiency of China’s coal chemical enterprises [20].

When using DEA, researchers usually take labor, capital, and energy as inputs, the output value of the chemical industry as the expected output, and environmental pollutants as the unexpected output to build an evaluation model of green development efficiency of the chemical industry. These indicators do not consider the characteristics of the chemical industry, which has a great impact on water environment. Tony Allan proposed “Virtual Water”; Hoekstra et al. proposed the concept of “Water Footprint”. Grey water footprint refers to the volume of freshwater required to dilute certain pollutants on the basis of existing water quality standards and natural background concentration [21]. Given that water footprint and grey water footprint can better represent the water consumption and water pollution accounting of industries [22], they have been gradually incorporated into the evaluation framework of the green development efficiency of regional industries [23,24].

The spatial distribution and environmental risk of the chemical industry in the Yangtze River Economic Belt have always been hot areas of academic concern. For a long time, the spatial layout of the chemical industry in the Economic Belt has reflected two major factors: the proximity to raw materials and market. The chemical industry along the Yangtze River is mainly distributed in the areas of Shanghai and Jiangsu [25]. In recent years, the petrochemical industry had a trend of expansion along the river to the upstream. The environmental pollution load gradient also shifted to the middle and upper reaches, and the environmental risk increased [26,27]. Xiang et al. (2021) found that the spatial differentiation characteristics of green development efficiency of the chemical industry in the Yangtze River Economic Belt were obvious. Economic level, scientific and technological innovation, industrial structure, and industrial agglomeration are the main factors affecting the spatial differentiation of green development efficiency of the chemical industry in the Economic Belt. The impact of foreign investment intensity and environmental regulation is relatively weak [28]. Therefore, it is necessary to guide the chemical industry of the Yangtze River Economic Belt to gather in large coastal bases and raw material producing or consumption areas, improve the rate of chemical enterprises entering the park, and optimize the spatial layout of the chemical industry [29]. Some scholars also studied the negative effects of the development of the chemical industry on the ecological environment. Zhu Deming et al. (2006) showed that the development of the chemical industry along the Yangtze River in Jiangsu threatened the drinking water source and water supply safety [30]. Intensive chemical enterprises and unreasonable industrial layout along the Yangtze River Economic Belt have brought some potential environmental risks to the environmental protection of the Yangtze River Basin [31,32]. Dong et al. (2020) found that the division level of heavy chemical industry in the middle and upper reaches of the Yangtze River Economic Belt decreased, which promoted the decline of the regional pollution level [33].

The contribution of this research is mainly reflected in the following aspects. Firstly, it makes up for the industry characteristics that little considered the impact of the chemical industry on the water environment in previous studies. In this study, water footprint and grey water footprint are included in the green development efficiency measurement index system of the chemical industry, and the green development efficiency of the chemical industry is established by using DEA, which was calculated from 2002 to 2016 in the Yangtze River Economic Belt. Secondly, Dagum Gini coefficient and its decomposition method are used to decompose the regional differences of green development efficiency of the chemical industry in the Economic Belt. Lastly, the convergence characteristics of green development efficiency of the chemical industry in the Economic Belt and its upstream, middle, and lower reaches are tested with the coefficient of variation method and panel data regression model from three aspects, i.e., convergence, absolute convergence, and conditional absolute convergence.

## 3. Materials and Methods

### 3.1. Regional Overview

**Figure 1:**
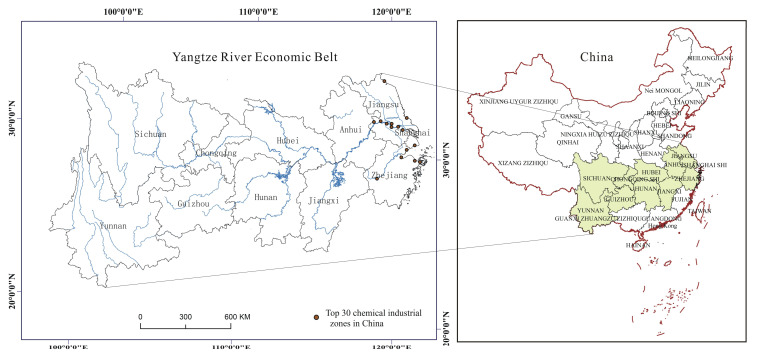
Map of the Yangtze Economic Belt.

The Yangtze River Economic Belt consists of the 11 provincial administrative units of Shanghai, Jiangsu, Zhejiang, Anhui, Jiangxi, Hubei, Hunan, Guizhou, Chongqing, Sichuan, and Yunnan (Figure 1) and covers an area of about 2.05 million km^2^, accounting for 21% of the country and more than 40% of the total population and economy [7]. It is one of the Chinese chemical industry agglomeration areas. In 2016, 11 provinces and cities in the Yangtze River Economic Belt achieved a total sales value of 8253.40 billion RMB, accounting for 43.46% of the total sales value in China. The sales output values of the chemical industry in downstream areas, middle reaches, and upstream area were 530.18 billion RMB, 1816.96 billion RMB, and 1134.663 billion RMB, accounting, respectively, for 27.91%, 9.57%, and 5.97% of that in China. The sales value of the chemical industry in Jiangsu province was the highest at 2954.89 billion RMB, about 15.56% of the whole country, while that in Yunnan province was the lowest at 139.00 billion RMB, accounting for 0.73% of that in the whole country [28].

### 3.2. Methods

#### 3.2.1. Calculation of Grey Water Footprint of the Chemical Industry

Industrial wastewater is directly discharged into surface water. The main pollutants in industrial wastewater can be measured directly, such as chemical oxygen demand (COD) and ammonia nitrogen (${\mathrm{NH}}_{4}^{+}$-N) in chemical industry wastewater. Therefore, COD and ${\mathrm{NH}}_{4}^{+}$-N are used as the main indicators to measure the grey water footprint of the chemical industry. The calculation formula is as follows [23]:

$$GWF_{ind}=\max(GWF_{ind(COD)},GWF_{ind(NH_{4}^{+}-N)})$$

$$GWF_{ind(i)}=\frac{L_{ind(i)}}{C_{\max}^{}-C_{nat}}-W_{ed}$$

$$GWF_{reg}=\sum_{i=1}^{n}GWF_{ind(i)}$$

where *GWF_ind_* (billion m^3^) is the grey water footprint of the chemical industry, *GWF_ind(i)_* (billion m^3^) is the grey water footprint of the chemical industry with the standard of category *i* pollutants, *W_ed_* (billion m^3^) is the discharge amount of chemical industry wastewater, and *GWF_reg_* (billion m^3^) is the grey water footprint of the regional chemical industry. China’s Standard Limits for Basic Items of Surface Water Environmental Quality Standard (GB 3838-2002) is used as the standard. In the standard, the water quality is required to meet the class III water quality index, and the concentration limits of COD and ammonia nitrogen (${\mathrm{NH}}_{4}^{+}$-N) in class III water are taken as the environmental concentration standards of COD and ammonia nitrogen (${\mathrm{NH}}_{4}^{+}$-N) in water.

#### 3.2.2. Measurement Model of Green Development Efficiency of the Chemical Industry

The SBM–undesirable model was proposed to measure the green development efficiency of the chemical industry. It is calculated as [34]: Supposing there are *n* individual *DMUs*, including input vector, expected output, and unexpected output, respectively, that are recorded as *x*, $x\in R_{m}^{}$, $y^{g}\in R_{s1}^{}$, and $y^{b}\in R_{s2}^{}$. The matrix is defined as

$$X=\left[x_{1},x_{2},\ldots ,x_{n}\right]\in R_{m\times n},\;Y^{g}=\left[y_{1}^{g},y_{2}^{g},\ldots ,y_{n}^{g}\right]\in R_{s1\times n},\;Y^{b}=\left[y_{1}^{b},y_{2}^{b},\ldots ,y_{n}^{b}\right]\in R_{s2\times n}$$

According to the actual input and output, supposing *x_i_* > 0, $y_{i}^{g}$ > 0, $y_{i}^{b}$ > 0, productive collection *P*, that is, *N* element input *X*. All combinations of expected and undesired outputs can be defined as

$$P=\left\{(x,y^{g},y^{b})\left|x\geq X\lambda ,y^{g}\geq Y^{b}\lambda ,y^{b}\geq Y^{b}\lambda (\lambda \geq 0)\right.\right\}$$

Therefore, the SBM–undesirable model can be expressed as

$$p*=\min\frac{1-\frac{1}{m}\sum_{i=1}^{i}\frac{S_{i}^{-}}{X_{i0}}}{1+\frac{1}{S_{1}+S_{2}}\left(\sum_{r=1}^{S_{1}}\frac{S_{r}^{g}}{y_{r0}^{g}}+\sum_{r=1}^{S_{2}}\frac{S_{r}^{b}}{y_{r0}^{b}}\right)},\;s.t.\left\{\begin{array}{l} X_{0}=X\lambda +S^{-}\\ y_{0}^{g}=Y^{{}_{g}}\lambda +S^{{}_{{}^{g}}}\\ y_{0}^{b}=Y^{b}\lambda +S^{b}\\ S^{-}\geq 0,S^{g}\geq 0,S^{b}\geq 0,\lambda \geq 0 \end{array}\right.$$

Type: $S_{i}^{-}$, $S_{r}^{g}$, and $S_{r}^{b}$, respectively, represent the first *i_0_* input redundancy, expected output deficiency, and expected output superscalar of each decision-making unit; $S_{i}^{-}$, $S_{r}^{g}$, and $S_{r}^{b}$, respectively, denote their corresponding vectors; and λ is the weight vector. The optimal solution of the above formula is (λ*, *S*^−^*, S^g^*, S^b^*). P* = 1 only when the bad output exists, that is, *S*^−^* = 0, S^g^* = 0, S^b^* = 0 when *DMU_0_* is efficient.

#### 3.2.3. Dagum Gini Coefficient and Decomposition Method

By using the Dagum Gini coefficient method, this study analyzes the spatial differences and sources of green development efficiency of the chemical industry in the upper, middle, and lower reaches of the Yangtze River Economic Belt. According to the Gini coefficient and its subgroup decomposition method proposed by Dagum (1997), the definition of Gini coefficient G is as shown in Equation (1) [35]:

(1) $$G=\frac{\sum_{j-1}^{k}\sum_{h-1}^{k}\sum_{i-1}^{n_{j}}\sum_{r-1}^{n_{h}}\left|y_{ji}-y_{hr}\right|}{2n^{2}\bar{y}}$$

where *j* and *h* are subscripts for different regions; *i* and *r* are the indexes of provinces and cities, respectively; *n* is the total number of provinces and cities; *k* is the total number of regions; and *n_j_*(*n_h_*) and *j*(*h*) are the number of provinces and cities within a region. *y_ji_(y_hr_)* is the green development efficiency of the chemical industry in *j(h)* regional provinces and cities *i(r)*, and $\bar{y}$ is the average value of green development efficiency of the chemical industry in all provinces and cities. On the overall Gini coefficient *G* by region, according to the average value of green development efficiency of the chemical industry in each region *k*, the region is sorted and then the Gini coefficient *G* is divided into three parts: intraregion (intra-group) difference pairs *G* contribution of *G_w_*, interregional (inter-group) difference pairs *G* contribution of *G_nb_*, and interregional (inter-group) ultra-variable density pairs *G* contribution of *G_t_*. When the three meet, *G* = *G_w_* + *G_nb_* + *G_t_*, in which the area *j* has a Gini coefficient of *G_jj_* and intraregional differences *G_w_*. The calculation formulas are Formulas (2) and (3), respectively; zones *j* and *h* have a Gini coefficient between *G_jh_* and the regional net difference *G_nb_*. The calculation formulas are Formulas (4) and (5), respectively. The calculation formula for the interregional super-variable density *G_t_* is shown in Formula (6).

(2) $$G_{jj}=\frac{\frac{1}{2{\bar{y}}_{j}}\sum_{i=1}^{n_{j}}\sum_{r=1}^{n_{j}}\left|y_{ji}-y_{jr}\right|}{n_{j}^{2}}$$

(3) $$G_{w}=\sum_{j-1}^{k}G_{jj}P_{j}S_{j}$$

(4) $$G_{jh}=\sum_{i=1}^{n_{j}}\sum_{r=1}^{n_{h}}\frac{\left|y_{ji}-y_{hr}\right|}{n_{j}n_{h}({\bar{y}}_{j}+{\bar{y}}_{h})}$$

(5) $$G_{nb}=\sum_{j-2}^{k}\sum_{h-1}^{j-1}G_{jh}(p_{j}s_{h}+p_{h}s_{j})D_{jh}$$

(6) $$G_{t}=\sum_{j=2}^{k}\sum_{h=1}^{j-1}G_{jh}(p_{j}s_{h}+p_{h}s_{j})(1-D_{jh})$$

In Equation (5), *p_j_* = *n_j_/n*, $s_{j}=n_{j}{\bar{y}}_{j}/n\bar{y}$, and *j* = 1, 2, 3. In Equation (7), *D_jh_* denotes region *j* and *h*. See Formula (7) for the relative influence of green development efficiency of the chemical industry. *d_jh_* is the difference of the green development efficiency of the chemical industry between regions (see Equation (8)). *j*, *h* all *y_ji_ − y_hr_* > 0 is the mathematical expectation of the sample summation; *p_jh_* is the super-variable first-order moment, representing the region. *j*, *h* all *y_hr_ − y_ji_* > 0 is the mathematical expectation of the sample summation.

(7) $$D_{jh}=\frac{d_{jh}-p_{jh}}{d_{jh}+p_{jh}}$$

(8) $$d_{jh}=\int_{0}^{\infty }dF_{j}(y)\int_{0}^{y}\left(y-x)dF_{h}(x\right)$$

(9) $$p_{jh}=\int_{0}^{\infty }dF_{h}(y)\int_{0}^{y}\left(y-x)dF_{j}(x\right)$$

where *F_j_(F_h_)* represents the area *j(h)C*, which is the cumulative distribution function of green development efficiency of the chemical industry.

#### 3.2.4. Convergence Model

To investigate the evolution trend of green development efficiency of the chemical industry in the whole Yangtze River Economic Belt and the upper, middle, and lower reaches, the convergence analysis is carried out, including σ Convergence and β Convergence.

σ Convergence refers to the trend where the deviation of green development efficiency of the chemical industry in different regions is decreasing over time. σ Convergence is measured by the coefficient of variation and can be calculated as [36]:

σ=∑iNj(Fij−F¯ij)2/NjF¯ij

where *j* indicates the number of areas (*j* = 1, 2, 3…), *i* indicates the number of provinces and cities in the region (*i* = 1, 2, 3…), *N_j_* is the number of provinces and cities in each region, and *F_ij_* denotes that the region *j* exists *t* with an average value of green development efficiency of the chemical industry in the period.

The β convergence model is [36]:

$$\ln(\frac{F_{i,t+1}}{F_{i,t}})=\alpha +\beta F_{i,t}+\mu_{i}+\nu_{t}+\varepsilon_{it}$$

The left side of the model is the growth rate of green development efficiency of the chemical industry calculated by logarithmic difference, where μ*_i_* is a fixed effect, *v_t_* is a time-fixed effect, and ε*_it_* is a random error term.

In condition β, the convergence model is absolute β. A series of control variables is added to the convergence model. This study adds environmental regulation, industrial structure, technical level, and foreign investment intensity as control variables. The convergence model for condition β is

$$\ln(\frac{F_{i,t+1}}{F_{i,t}})=\alpha +\beta F_{i,t}+\delta X+\mu_{i}+\nu_{t}+\varepsilon_{it}$$

In the regression process, each variable is logarithmic. In this paper, a two-way fixed effect model is adopted to improve the coefficient. In the β accuracy of estimation, the robust error standard of clustering is adopted to the provincial and municipal levels. If β < 0 and is significant, the green development efficiency of the chemical industry in the Yangtze River Economic Belt converges, or it diverges. The rate of convergence *b* = −ln(1+ β)/*T*.

### 3.3. Index Selection and Data Processing

#### 3.3.1. Measurement Index of Green Development Efficiency of the Chemical Industry

According to existing research results, combined with the classification and characteristics of the chemical industry, the evaluation index system of green development efficiency of the chemical industry is constructed from input and output. Manpower, capital, energy, and water for the chemical industry are selected as investment indexes. The sales output value of the chemical industry is selected as the expected output index and the grey water footprint of the chemical industry as the unexpected output index (Table 1).

Considering the availability of data of the chemical industry, the scope of the chemical industry is defined as five subsectors in the manufacturing industry by the *China Industrial Statistics Yearbook*: petroleum processing, coking and nuclear fuel processing; chemical raw materials and chemical products manufacturing; pharmaceutical manufacturing; chemical fiber manufacturing; and rubber and plastic products manufacturing. Relevant data come from the *China Industrial Statistics Yearbook*, *China Environmental Statistics Yearbook*, *China Statistical Yearbook*, and statistical yearbooks of various provinces and cities from 2003 to 2017. *China Industrial Statistical Yearbook*, *China Environmental Statistical Yearbook*, and *China Statistical Yearbook* are the most authoritative and important sources of data for conducting research on China’s socioeconomic development, available in both paper and electronic versions, published annually by the National Bureau of Statistics of China, and can be accessed through a variety of official channels for direct access to relevant data. The details are as follows. The missing data are estimated by intermediate interpolation method. There are no direct statistical data of total industrial water consumption and wastewater discharge in the statistical yearbook, so we apply the data of industrial wastewater and pollutant discharge in wastewater for each subsector in China to estimate the data of pollutant discharge in industrial wastewater for each subsector in each province [37]. Energy data of the chemical industry are estimated by reference [38]. For the net fixed capital and industrial sales output value of the chemical industry, the fixed assets investment price index of corresponding provinces and cities and the ex-factory price index of industrial producers are used for price reduction, which is reduced to the level of 2000.

#### 3.3.2. Variables Affecting the Efficiency of Green Development of the Chemical Industry

Using environmental regulations, industrial structure, foreign investment intensity and technological progress as control variables, this paper studied their influence on the green development efficiency of the chemical industry in the Yangtze River Economic Belt. Among them, the total amount of environmental governance for environmental regulation represents the proportion of GDP. Science and technology investment is represented by the proportion of science and technology expenditure in fiscal expenditure, which is representative of the investment amount of foreign-funded enterprises at the end of the year. The proportion of the secondary industry in GDP represents the industrial structure.

## 4. Results

### 4.1. Evolution Characteristics of Grey Water Footprint of the Chemical Industry in the Yangtze River Economic Belt

The grey water footprint of the chemical industry in the Yangtze River Economic Belt declined from 2002 to 2016 with a trend of fluctuation. It decreased from 16.03 billion m^3^ in 2002 to 12.03 billion m^3^ in 2008 and then increased to 14.43 billion m^3^ in 2016. In 2008, due to the impact of the financial crisis, the operation of chemical enterprises was impacted, the production capacity decreased, and the total grey water footprint was at its lowest point. After the financial crisis, thanks to the support of relevant national policies, chemical enterprises gradually eliminated the crisis, the output of the chemical industry gradually recovered, and the discharge of wastewater in the chemical industry increased, leading to an increase in the total grey water footprint of the chemical industry.

**Figure 2:**
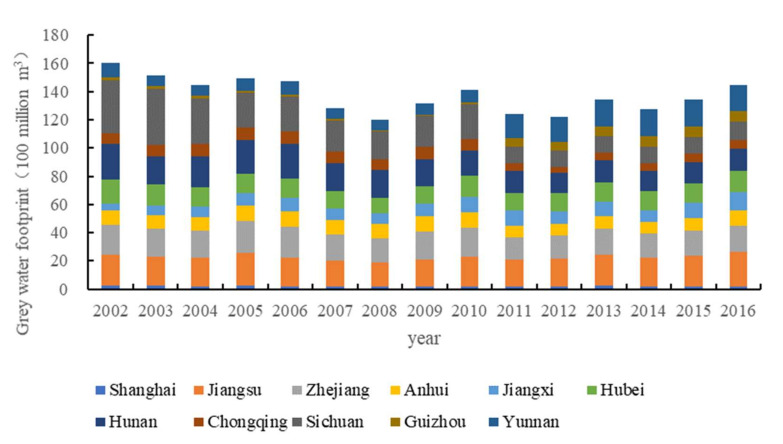
Grey water footprint of the chemical industry in Yangtze River Economic Belt.

The grey water footprint of the chemical industry in Jiangsu, Zhejiang, Hubei, Hunan, Sichuan, and Yunnan provinces is relatively high. These provinces are the main concentration provinces of the chemical industry in the Economic Belt, with large-scale enterprises that have high amounts of wastewater discharge. The chemical industry in Shanghai has the lowest wastewater footprint. On the one hand, Shanghai has accelerated the adjustment of its industrial structure, the proportion of the chemical industry in the national economy has decreased, and the overall scale of the chemical industry has shrunk. In 2016, the sales value of its chemical industry only accounted for 2.67% of that in China. On the other hand, the chemical industry in Shanghai is gradually transforming and upgrading to the direction of a high-end, green, and low-carbon chemical industry. Shanghai has carried out the construction of a “Green Industrial zone” earlier in China, and its environmental and economic indicators of 10,000 CNY of output value led the national level of the same industry. The grey water footprint of the chemical industry in Guizhou is relatively low, mainly because of the small scale of the chemical industry. In 2016, the sales value of the chemical industry in Guizhou only accounted for 0.83% of the national total (Figure 2).

### 4.2. Spatial and Temporal Evolution of Green Development Efficiency of the Chemical Industry in Yangtze River Economic Belt

From 2002 to 2016, the green development efficiency of the chemical industry in the Yangtze River Economic Belt showed an overall development and evolution trend of first decreasing and then increasing, with an average of 0.5163, only reaching the optimal level of 51.63% (Table 2). This trend showed that the overall level of green development efficiency of the chemical industry is not high and still has great growth potential. Note that the green development efficiency of the chemical industry showed a downward trend from 2002 to 2005, which may be due to the reversal of China’s economic model in the later stage of its 11th Five-Year Plan. Moreover, the chemical industry turned back to the development model of high consumption, high pollution emission, and low efficiency. The average green development efficiency of the chemical industry in the Yangtze River Economic Belt increased significantly during 2012 and 2016, which is the reason that provinces and cities in the Economic Belt accelerated the green development, transformation, and upgrading of the chemical industry and achieved remarkable results after the 18th National Congress.

From the upstream, midstream, and downstream areas, the average green development efficiency from 2002 to 2016 of the chemical industry in the downstream area was 0.8343, which was in a high-level development state with a small overall change range. The average green efficiencies of the chemical industry in the midstream and upstream areas were 0.4156 and 0.2739, respectively, which are relatively low and generally show an evolutionary trend of first declining and then rising (Table 2).

**Figure 3:**
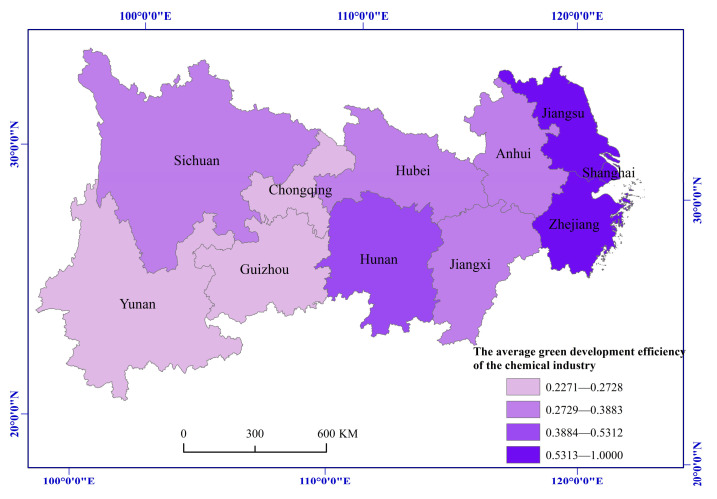
The average green development efficiency of the chemical industry from 2002 to 2016.

In terms of provinces and cities, the green development efficiency of the chemical industry in Shanghai, Zhejiang, and Jiangsu has been maintained at the optimal state of 1.00 (except when it was at 0.79 in 2005), while that in Hunan province also reached the optimal state of 1.00 from 2012 to 2016. The green development efficiency of the chemical industry in Anhui, Hubei, Chongqing, Sichuan, Guizhou, and Yunnan provinces increased to varying degrees, showing a development trend of first decreasing and then increasing. However, there is still a large gap in the green development efficiency of the chemical industry between these provinces and the Shanghai, Zhejiang, and Jiangsu provinces (Figure 3).

### 4.3. Regional Difference Analysis of Green Development Efficiency of the Chemical Industry in the Yangtze River Economic Belt

To further reveal the regional differences and sources of green development efficiency of the chemical industry in the Yangtze River Economic Belt, Dagum Gini coefficient and its decomposition method were used to calculate and decompose its relative level.

#### 4.3.1. Overall Regional Differences

From 2002 to 2016, the average regional difference of green development efficiency of the chemical industry in the Yangtze River Economic Belt was 0.3080, showing a development trend of first expanding and then narrowing. The maximum and minimum regional differences of green development efficiency of the chemical industry appeared in 2007 and 2016 at 0.3347 and 0.2577, respectively. From 2007 to 2010, the green development efficiency of the chemical industry fluctuated greatly, due mainly to the impact of the financial crisis and the inconsistent degree of recovery of such efficiency in various provinces and cities. From 2012 to 2016, the regional differences in green development efficiency of the chemical industry narrowed, mainly because since the 18th National Congress of the Communist Party of China, the middle and upper reaches with low green development efficiency of the chemical industry have strengthened the treatment of the chemical industry, improved its resource and energy utilization efficiency, reduced waste water discharge, and improved the green development efficiency, thereby reducing the regional differences in green development efficiency of the chemical industry.

#### 4.3.2. Intraregional Differences

On the whole, the regional difference of green development efficiency of the chemical industry in downstream areas is the largest, with an average of 0.1472. The second is the middle reaches, with an average of 0.1043. The upstream area is the smallest, with an average of 0.0873. From the evolution trend, from 2002 to 2012, the regional difference of green development efficiency of the chemical industry in the middle and lower reaches showed an upward trend in fluctuation. From 2012 to 2016, it showed a downward trend. From 2002 to 2016, the green development efficiency of the chemical industry in the upstream region showed an upward trend in fluctuation, indicating that the regional differences are expanding. Note that, although the regional differences of green development efficiency of the chemical industry in the upstream region is the smallest, they are expanding. Hence, it is necessary to strengthen the regulation of regional difference of green development efficiency of the chemical industry in the upstream region. Although there are great regional differences in the green development efficiency of the chemical industry in the middle reaches and downstream areas, these have narrowed significantly since 2012.

#### 4.3.3. Differences between Regions

**Figure 4:**
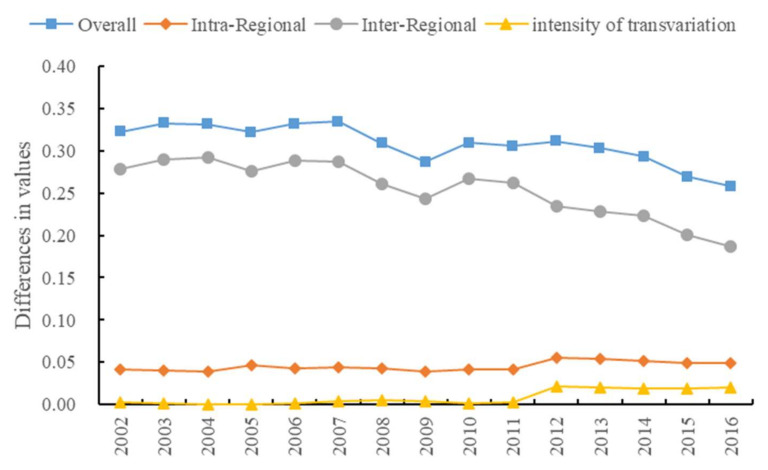
Regional differences in green development performance of the chemical industry in the Yangtze River Economic Belt.

From the mean value of green development efficiency of the chemical industry among the three regions, the regional difference between the downstream and upstream areas is the largest at 0.5081, between the lower and middle reaches is 0.3805, and between the middle reaches and the upper reaches is the smallest at 0.1872. From the changing trend, the regional difference between the middle and upper reaches tends to expand, whereas those between downstream and upstream and between downstream and midstream tend to narrow (Figure 4).

#### 4.3.4. Source of Difference

From the perspective of difference sources, the contribution of inter-group differences is the largest with an average value of 0.2545, which is higher than that of regional differences with an average value of 0.0453, and the contribution of over variable density with an average value of 0.0082. The evolution trend of inter-group differences is similar to that of overall regional differences, and the contribution rate of the average value of inter-group differences is as high as 78.94%. This percentage showed that the difference between groups is the main factor affecting the overall regional difference of green development efficiency of the chemical industry in the Yangtze River Economic Belt. The contribution rates of intra-group difference and hypervariable density were 14.05% and 2.54%, respectively, which have relatively small contributions to the overall regional difference (Table 3).

### 4.4. Regional Convergence Analysis of Green Development Efficiency of the Chemical Industry in the Yangtze River Economic Belt

#### 4.4.1. σ-Convergence Test

**Figure 5:**
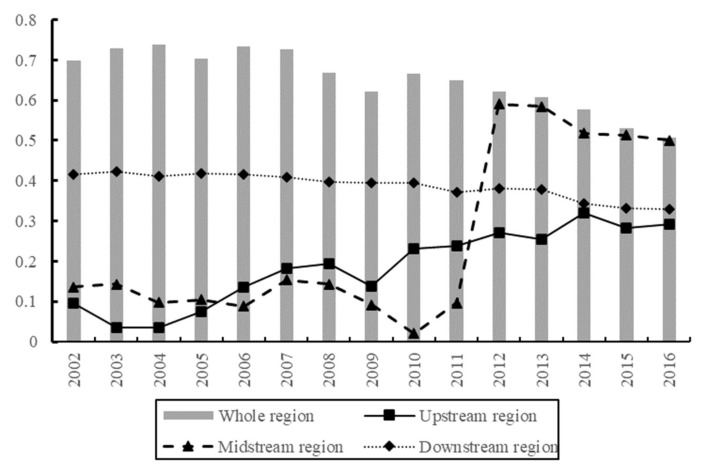
Absolute convergence graph.

The σ-convergence test method is used to calculate the σ-convergence coefficient of the whole Yangtze River Economic Belt and the upper, middle, and lower reaches, as shown in Figure 5. From 2002 to 2004, the global σ-convergence coefficient of the Economic Belt increased and showed a divergent state. From 2006 to 2016, the global σ-convergence coefficient generally showed a downward trend, indicating that the global σ-convergence occurred. This means that the regional differences in the green development efficiency of the chemical industry in the Yangtze River Economic Belt are shrinking.

From different regions, the σ-convergence coefficient of downstream areas showed a downward trend from 2002 to 2016 and σ-convergence, indicating that the regional difference in green development efficiency of the chemical industry in downstream areas had narrowed. From 2002 to 2012, the σ-convergence coefficient in the middle reaches increased and then decreased, and after 2012, it continued to decline, showing σ-convergence. After 2012, the regional difference of green development efficiency of the chemical industry in the middle reaches was reduced. From 2002 to 2016, the σ-convergence coefficient in the upstream region basically showed an upward trend and no σ-convergence, indicating that the difference in green development efficiency of the chemical industry in the upstream region was expanding.

Overall, the green development efficiency of the chemical industry in the whole region and the downstream areas of the Economic Belt has σ-convergence. After 2012 in the middle reaches, the green development efficiency of the chemical industry also had σ-convergence. There is no σ-convergence in the upstream region, and the regional imbalance of green development efficiency of the chemical industry intensified, which is basically consistent with the analysis results of Gini coefficient.

#### 4.4.2. Absolute Convergence of β

The Hausman test shows that the panel data model with time and individual double fixed effects is more appropriate, and so the β absolute convergence mechanism was tested. The results show that the β absolute convergence coefficients in the whole region and the upper, middle, and lower reaches of the Yangtze River Economic Belt are negative, indicating that the green development efficiency of its chemical industry exists in β absolute convergence. Among them, the significance of the whole region, the upstream region, and the middle reaches region passed the significance test of 1%, 5%, and 5%, respectively. These results showed that the growth rate of green development efficiency of the chemical industry in the whole region, the upstream region, and the middle reaches region of the Yangtze River Economic Belt converged, while the significance of the downstream region did not pass the test. In terms of convergence speed, the upstream region is the fastest, followed by the midstream region (Table 4).

Conditional β convergence does not require different regions to have the same basic characteristics, i.e., different regions can be at different growth paths and steady-state levels. If conditional β convergence exists, they eventually converge to their respective steady states by virtue of their own characteristics. In this paper, the green development efficiency of chemical industry in the Yangtze River Economic Belt is examined in four aspects, namely environmental regulation, industrial structure, foreign investment intensity, and scientific and technological progress, to investigate which factors contribute to the green development efficiency of chemical industry in Yangtze River Economic Belt to reach the conditional convergence. After controlling the control variables, such as environmental regulation, industrial structure, foreign capital intensity, and scientific and technological progress, the β absolute convergence coefficient in the whole region and the upper, middle, and lower reaches of the Yangtze River Economic Belt was still negative. In addition, the significance of the whole region and the middle reaches passed the significance test at 1% and 5%, respectively. This shows that the green development efficiency of the abovementioned regional chemical industry follows the trend of β absolute convergence, which is under the consideration of environmental regulation, industrial structure, foreign capital intensity, and scientific and technological progress. In terms of convergence rate, the convergence rate in the middle reaches is faster.

In the panel data regression model of the whole region and the upper, middle, and lower reaches of the Yangtze River Economic Belt, the regression coefficients of the control variable environmental regulation are negative, and the whole region and the upper reaches pass the 5% and 10% significance tests, respectively. This finding showed that the environmental regulation of the whole region and the upper reaches restricts the reduction in regional difference in the green development efficiency of the chemical industry. The regression coefficients of industrial structure in the whole region, the middle reaches, and the downstream regions are positive, while those of the upstream regions are negative, but they all fail to pass the significance test. The regression coefficient of foreign capital intensity in the whole region, the upstream region, and the downstream region is positive, while that of the middle reach region is negative, but only the upstream region passes the significance test. The results showed that the foreign capital intensity helps reduce the regional difference of green development efficiency of the chemical industry in the upstream region. The regression coefficients of scientific and technological progress in the whole region, upstream, midstream, and downstream regions are positive, but only the whole region passes the significance test. The outcome means that the improvement of scientific and technological level helps reduces the difference in green development efficiency of the chemical industry in these regions but is not the main reason (Table 5).

## 5. Discussion

First, the problem of water ecological environment in the Yangtze River Economic Belt has attracted increasing research attention. Grey water footprint has been widely recognized as an indicator of pollution intensity [39,40]. The industrial grey water footprint index can better reflect the water pollution of industrial production activities than the wastewater pollutant discharge index can [23]. In this study, grey water footprint is incorporated into the evaluation framework of green development efficiency of the chemical industry. It can better reflect the impact of chemical industry production activities on the water environment and provide new research ideas for the calculation of green development efficiency of the chemical industry. In recent years, the green development, transformation, and upgrading of the chemical industry in the Economic Belt has achieved remarkable results. Nonetheless, in the future, we should still focus on the dynamic change trend of pollutant discharge in chemical industry wastewater, further strengthen the water pollution control of the chemical industry, optimize the industrial scale, reduce the grey water footprint of the chemical industry, and improve the efficiency of grey water footprint of the chemical industry. On the basis of this calculation model, provinces and cities can also build a measurement model of green development efficiency of the chemical industry based on grey water footprint, monitor the green development of the chemical industry, and put forward governance strategies. However, the water consumption and grey water footprint data of the chemical industry used in this paper were estimated using the provincial and industrial data in the *Yearbook of China Economic Census* (2008) and reference [38], and there is a certain error with the actual value. In future research, it is still necessary to improve the accuracy of data estimation or expand the channels for obtaining water environment data of the chemical industry.

Second, this study showed that the green development efficiency of the chemical industry in the Yangtze River Economic Belt increased significantly from 2002 to 2016 and showed a development and evolution trend of first declining and then rising. Yijun Zhang (2020) found that the overall green development performance of China’s chemical industry showed a significant improvement trend from 2007 to 2017 [6]. Yeh Jiahuey (2019) studied the green development performance of China’s chemical industry from 1980 to 2013 and found that it showed an evolution law of first declining and then rising from 2002 to 2013 [17]. These results are basically consistent with the results of the present research. In recent years, the Chinese government has strengthened the construction of ecological civilization and actively promoted the green transformation and upgrading of the chemical industry with remarkable results. In particular, the 18th Congress of the Communist Party of China in 2012 proposed to give prominence to the construction of ecological civilization and build a “Beautiful China.” According to the data of the China Development and Reform Commission from 2016 to 2020, there were more than 8000 chemical enterprises along the Yangtze River. Furthermore, the proportion of excellent water environment sections in the Yangtze River Basin increased from 82.3% in 2016 to 91.7% in 2019 and further increased to 96.3% from January to November in 2020, and the proportion of inferior class V water quality in the Yangtze River Basin decreased from 3.5% to 0.6% during 2016 and 2019. The elimination of inferior class V water bodies would be realized for the first time in 2020, which showed that the green development of the chemical industry in the Economic Belt has achieved remarkable results, thus supporting the conclusions of this paper from the practical level.

Third, the regional heterogeneity of green development efficiency of the chemical industry in the Yangtze River Economic Belt is very obvious. Affected by the natural geographical environment and socioeconomic conditions, there are obvious gaps in the socioeconomic development level, industrialization level, and scientific and technological innovation ability in the upper, middle, and lower reaches of the Yangtze River Economic Belt. The zonality of industrial ecological efficiency [41] and green development level [42] is significant. This study found that the green development efficiency of the chemical industry in the Yangtze River Economic Belt was the highest in the lower reaches from 2002 to 2016, followed by the middle reaches, and the lowest in the upper reaches. The overall regional difference and interregional difference tended to narrow, and the intraregional difference expanded. Our result is similar to the research conclusions of Yunbo Xiang (2021) on the spatial differences of green development efficiency of the chemical industry in the Yangtze River Economic Belt [28], but there are some differences in specific values, which may be caused by different measurement indicators and used models. According to the above research results, in the future, the focus of the chemical industry governance in the Yangtze River Economic Belt would still be to accelerate the green development, transformation, and upgrading of the chemical industry in the middle and upper reaches, improve the green development efficiency of the chemical industry, and reduce regional differences. At the same time, we should pay attention to controlling regional differences and preventing their expansion. The middle and upper reaches should improve the green development efficiency of the chemical industry by means of technological innovation, optimizing the industrial scale, adjusting industrial structure, and strengthening environmental regulation. At the same time, we should promote the diffusion of technology, capital, and talents in the lower reaches to the middle and upper reaches. We should promote the green and coordinated development of the chemical industry in the Yangtze River Economic Belt.

## 6. Conclusions

This research studied the regional differences, influencing factors, and convergence of green development efficiency of the chemical industry in the Yangtze River Economic Belt by using the super efficiency SBM model, Dagum Gini coefficient, coefficient of variation method, and panel data regression model. The green development efficiency measurement model of the chemical industry constructed in this paper can more objectively and comprehensively reflect the impact of the chemical industry on the water environment than in previous studies and is very consistent with the industrial characteristics of the chemical industry and the regional water environment problems of the Economic Belt. Accurately measuring the green development efficiency of the chemical industry in the Economic Belt can provide theoretical support for chemical water treatment and green development in the area. Analyzing the difference, influence, and convergence of green development efficiency of the chemical industry in the Yangtze River Economic Belt can provide reference for formulating strategies to improve the green development efficiency of the chemical industry by region and classification.

The main conclusions of this paper are as follows.

First, from 2002 to 2016, the total grey water footprint of the chemical industry in the Yangtze River Economic Belt showed a downward trend, while the green development efficiency of the chemical industry showed an upward trend as a whole. This finding means that remarkable achievements have been made in environmental governance and green development of the chemical industry in the Yangtze River Economic Belt in recent years.

Second, there are significant regional differences in the green development efficiency of the chemical industry in the Yangtze River Economic Belt. From the perspective of space, the green development efficiency of the chemical industry in the lower reaches is high, while that in the middle and upper reaches is low. In terms of time, the overall regional differences and interregional differences tend to narrow, and the intraregional differences expand. From the source of difference, regional difference is the main source of regional difference in green development efficiency of the chemical industry in the Yangtze River Economic Belt.

Third, there is σ-convergence in the green development efficiency of the chemical industry in the whole region, the middle reaches, and the lower reaches of the Yangtze River Economic Belt, while there is no σ-convergence in the upper reaches. There are β absolute convergence and β conditional convergence in the green development efficiency of the chemical industry in the whole region and the upper, middle, and lower reaches of the Yangtze River Economic Belt.

Fourth, there is spatial heterogeneity in the impact of environmental regulation, industrial structure, foreign capital intensity, and scientific and technological progress on the green development efficiency of the chemical industry in the Yangtze River Economic Belt. This spatial heterogeneity suggests that in the governance of the chemical industry in the Yangtze River Economic Belt, common but differentiated policy measures should be formulated and precisely applied according to the characteristics of the region, by region and by industry. The middle and upper reaches should strengthen environmental regulation, adjust the structure of chemical industry, strengthen supervision and management of foreign investment, and improve environmental access standards. The lower reaches should focus on scientific and technological innovation, promote the upgrading of chemical industry value chain, and enhance the resilience of chemical industry.

It should be noted that, due to the limitation of statistical data, the paper uses the proportion of COD and ammonia nitrogen (${\mathrm{NH}}_{4}^{+}$-N) emissions in industrial wastewater of the chemical industry by industry nationwide to estimate the COD and ammonia nitrogen (${\mathrm{NH}}_{4}^{+}$-N) emissions in wastewater of the chemical industry by industry in each province when calculating the gray water footprint, without distinguishing the proportion of COD and ammonia nitrogen (${\mathrm{NH}}_{4}^{+}$-N) emissions in wastewater between provinces and regions differences. To some extent, this underestimates the differences in gray water footprints of chemical industries in the Yangtze River Economic Belt among provinces and cities. Meanwhile, the study of the differences in the overall green development efficiency of the chemical industry in the Yangtze River Economic Belt and its convergence can help to grasp the green development efficiency of the chemical industry in provinces and cities in general, but in-depth studies on the gray water footprint and green development efficiency of five subsectors are needed in the future to reveal the differences between industries and to develop more refined governance strategies for the chemical industry.

## Figures and Tables

**Table 1:** Evaluation index system of green development efficiency of the chemical industry.

Index		Variable	Variable Declaration
Input index		Human input	Average annual number of employees in chemical industry (10,000)
		Capital input	Net fixed assets of chemical industry (100 million CNY)
		Energy input	Total energy consumption of chemical industry (ten thousand tec)
		Water input	Chemical industry water consumption (100 million m^3^)
Output index	Expected output	Chemical industry output value	Sales output value of chemical industry (100 million CNY)
	Unexpected output	Water pollution	Chemical industry grey water footprint (billion m^3^)

**Table 2:** Green development efficiency of the chemical industry in the Yangtze River Economic Belt from 2002 to 2016.

	2002	2004	2006	2008	2010	2012	2014	2016 Year	Average
Guizhou	0.2809	0.2351	0.2131	0.2176	0.1984	0.1916	0.1973	0.2612	0.2271
Sichuan	0.2390	0.2538	0.2836	0.3366	0.3461	0.3634	0.4109	0.4922	0.3387
Yunnan	0.2243	0.2401	0.2581	0.3114	0.2640	0.2395	0.2422	0.2916	0.2569
Chongqing	0.2508	0.2370	0.2204	0.2526	0.2517	0.2742	0.3041	0.3918	0.2728
Hubei	0.3794	0.3117	0.3173	0.3687	0.3447	0.4368	0.4817	0.5043	0.3883
Hunan	0.2938	0.2609	0.2718	0.3177	0.3363	1.0000	1.0000	1.0000	0.5312
Jiangxi	0.3132	0.2679	0.2746	0.2770	0.3506	0.3521	0.4005	0.4052	0.3273
Anhui	0.3129	0.3177	0.3099	0.3357	0.3410	0.3593	0.4124	0.4346	0.3512
Jiangsu	1.0000	1.0000	1.0000	1.0000	1.0000	1.0000	1.0000	1.0000	0.9860
Shanghai	1.0000	1.0000	1.0000	1.0000	1.0000	1.0000	1.0000	1.0000	1.0000
Zhejiang	1.0000	1.0000	1.0000	1.0000	1.0000	1.0000	1.0000	1.0000	1.0000
Upstream area	0.2488	0.2415	0.2438	0.2795	0.2650	0.2672	0.2886	0.3592	0.2739
Midstream area	0.3288	0.2802	0.2879	0.3211	0.3438	0.5963	0.6274	0.6365	0.4156
Downstream area	0.8282	0.8294	0.8275	0.8339	0.8352	0.8398	0.8531	0.8586	0.8343
Whole area	0.4813	0.4658	0.4681	0.4925	0.4939	0.5652	0.5863	0.6164	0.5163

**Table 3:** Regional differences in green development efficiency of the chemical industry in the Yangtze River Economic Belt.

Year	Overall G	Inter Group	Between Groups	Hypervariable Density	Upstream	Midstream	Downstream	Midstream– Upstream	Downstream– Upstream	Downstream– Midstream
2002	0.3224	0.0415	0.2786	0.0023	0.0456	0.0579	0.1556	0.1386	0.5380	0.4413
2003	0.3327	0.0408	0.2900	0.0019	0.0151	0.0597	0.1588	0.1264	0.5540	0.4677
2004	0.3312	0.0392	0.2921	0.0000	0.0153	0.0403	0.1542	0.0741	0.5490	0.4950
2005	0.3220	0.0463	0.2753	0.0003	0.0350	0.0419	0.1829	0.0831	0.5239	0.4621
2006	0.3321	0.0426	0.2885	0.0010	0.0638	0.0351	0.1564	0.0894	0.5448	0.4849
2007	0.3347	0.0446	0.2867	0.0034	0.0832	0.0679	0.1531	0.1058	0.5411	0.4848
2008	0.3089	0.0435	0.2605	0.0049	0.0930	0.0634	0.1494	0.1006	0.4980	0.4487
2009	0.2869	0.0389	0.2438	0.0042	0.0610	0.0405	0.1484	0.1051	0.4767	0.4070
2010	0.3093	0.0412	0.2672	0.0010	0.1074	0.0092	0.1479	0.1325	0.5188	0.4187
2011	0.3054	0.0414	0.2615	0.0025	0.1148	0.0381	0.1394	0.1211	0.5045	0.4253
2012	0.3110	0.0551	0.2345	0.0215	0.1287	0.2414	0.1430	0.3834	0.5178	0.2529
2013	0.3029	0.0541	0.2280	0.0208	0.1227	0.2369	0.1420	0.3654	0.5024	0.2503
2014	0.2933	0.0517	0.2228	0.0188	0.1522	0.2123	0.1291	0.3717	0.4944	0.2264
2015	0.2691	0.0494	0.2003	0.0194	0.1332	0.2122	0.1241	0.3174	0.4421	0.2233
2016	0.2577	0.0493	0.1875	0.0209	0.1380	0.2077	0.1235	0.2931	0.4160	0.2194

**Table 4:** β Absolute convergence table.

Variable	Whole Region	Upstream Region	Midstream Region	Downstream Region
β	−0.1734 ***	−0.2545 **	−0.1806 **	−0.2691
	(−4.43)	(−3.86)	(−7.13)	(−0.92)
Constant term	−0.1996 ***	−0.4009 **	−0.2761 **	−0.0877
	(−5.31)	(−4.46)	(−9.73)	(−1.31)
R^2^	0.0736	0.3676	0.4548	0.1027
Convergence rate	0.0127%	0.0196%	0.0132%	-

Note: ** and ***, respectively, represent significance at the confidence levels of 5% and 1%, and T statistics are in brackets. “-” means empty.

**Table 5:** β Conditional convergence table.

Variable	Whole Region	Upstream Region	Midstream Region	Downstream Region
β	−0.1564 ***	−0.2476	−0.1920 **	−0.3976
	(−3.61)	(−1.25)	(−8.18)	(−1.41)
Environmental regulation	−37.6551 **	−11.4104 *	−51.8642	−30.9161
	(−3.04)	(−2.43)	(−1.00)	(−1.01)
Industrial structure	0.3754	−0.4512	1.5735	0.3231
	(1.53)	(−1.93)	(1.02)	(2.01)
Foreign capital intensity	0.8206	7.6279 **	−5.4985	0.9576
	(1.26)	(4.49)	(−0.65)	(2.04)
Science and technology	1.7491 **	6.4771	2.5444	1.8611
	(2.89)	(1.08)	(0.99)	(1.30)
Constant term	−0.3230 ***	−0.3667	−0.6851	−0.3956
	(−3.72)	(−1.79)	(−1.44)	(−2.28)
R^2^	0.0250	0.0854	0.1260	0.0748
Convergence rate	0.0113%	-	0.0142%	-

Note: *, **, and ***, respectively, represent significance at the confidence levels of 10%, 5%, and 1%, and T statistics are in brackets. “-” means empty.

## Data Availability

The data presented in this study are available on request from the corresponding author.
